# No association between numerical ability and politically motivated reasoning in a large US probability sample

**DOI:** 10.1073/pnas.2301491120

**Published:** 2023-07-31

**Authors:** Michael N. Stagnaro, Ben M. Tappin, David G. Rand

**Affiliations:** ^a^Sloan School of Management, Massachusetts Institute of Technology, Cambridge, MA 02142

**Keywords:** motivated reasoning, polarization, decision-making, political psychology

## Abstract

The highly influential theory of “Motivated System 2 Reasoning” argues that analytical, deliberative (“System 2”) reasoning is hijacked by identity when considering ideologically charged issues—leading people who are more likely to engage in such reasoning to be more polarized, rather than more accurate. Here, we fail to replicate the key empirical support for this theory across five contentious issues, using a large gold-standard nationally representative probability sample of Americans. While participants were more accurate in evaluating a contingency table when the outcome aligned with their politics (even when controlling for prior beliefs), we find that participants with higher numeracy were more accurate in evaluating the contingency table, regardless of whether or not the table’s outcome aligned with their politics. These findings call for a reconsideration of the effect of identity on analytical reasoning.

How people reason about ideologically charged issues is hotly contested. The standard assumption that reasoning leads to more accurate beliefs has been challenged by the idea of (directionally) *motivated* reasoning (1). The general theory of motivated reasoning argues that, when an individual has a personal stake in reaching a particular conclusion, reasoning is clouded by bias and rendered less able to effectively use available evidence to form accurate beliefs. Instead, reasoning works as “a lawyer,” producing arguments and evaluating evidence to support the reasoner’s desired conclusion.

In recent years, an extension of the motivated reasoning thesis—known as “Motivated System 2 Reasoning” (MS2R; also called “Motivated Numeracy”) (2)—has become highly influential in academic and lay perceptions of motivated reasoning. This extended theory adopts a dual-process perspective on decision-making, in which individuals are assumed to vary in their reliance on intuitive, automatic (“System 1”) decision-making processes versus analytical, deliberative (“System 2”) reasoning. MS2R then argues that individuals who engage in more reasoning should be better at producing arguments and evidence to support their desired conclusion (and rejecting evidence that challenges their desired conclusion). As a result, on issues where there is strong ideological or cultural conflict, individuals who are most analytic are expected to be the most biased or polarized in their beliefs, as they are most effective at deploying their reasoning abilities to support the positions favored by their “side.”

The finding often described as providing the strongest empirical evidence for MS2R comes from a now-classic paradigm introduced by Kahan and colleagues (2, 3). Participants were provided with a description of a scientific study that examined either a politically charged topic (gun control) or a neutral topic (skin cream). Participants were then shown the results of this “study” in the form of a two-by-two contingency table and asked to interpret the table (i.e., to indicate what association was demonstrated in the table). The contingency table was constructed in such a way that participants needed to invest cognitive effort in numerical reasoning to reach the correct answer, and which answer was correct was randomized across participants.

When the correct answer was aligned with the participant’s identity commitments (or was nonpolitical), participants who scored higher on a numeracy test were more likely to interpret the contingency table correctly. Critically, however, when the correct answer ran counter to the participant’s identity commitments, the advantage conferred by numeracy disappeared: Those who scored higher on the numeracy test were no more likely to interpret the contingency table correctly than those who scored lower. As a result, political polarization in the interpretation of the data was highest among the most numerate individuals.

This surprising result has attracted a great deal of attention. It has been widely interpreted as evidence that people with superior reasoning abilities use them to defend their identities in the context of ideologically divisive topics—and, thus, that inaccurate beliefs about such topics are better explained by motivated reasoning than insufficient reasoning (e.g., in the context of susceptibility to misinformation). Notably, however, this result could also be explained by differences in individuals’ prior beliefs, where those who score higher on reasoning tasks are more deferential to their priors on a given topic rather than more deferential to their identities (4).

Beyond this conceptual issue, the replicability and generalizability of the key finding itself are unclear. While one independent team replicated the finding in an Australian sample (5), numerous others have failed to replicate it (6–11), and still others have found mixed results (12–14). Thus, the robustness of this central piece of evidence for MS2R is unclear.

The authors of the original study have argued that obtaining a large sample that accurately represents the distribution of “typical” US citizens is essential for identifying MS2R and have suggested that replication failures may be due to the use of small convenience samples rather than the probability sample of American participants used in the original work (3, 15).

Here, we assess this possibility by providing the first independent preregistered test of the *replicability* of the original finding using a gold-standard representative probability sample of Americans. Importantly, we conferred with one of the original authors from refs. 2 and 3 and confirmed that the AmeriSpeak panel provides sufficiently representative samples for this replication to satisfy earlier critiques regarding representativeness. Our sample is also twice as large as the original study, providing additional statistical power.

Furthermore, we assess the generalizability of the original finding by examining gun control as well as four additional topics that pretesting indicates are highly polarizing for both Democrats and Republicans. Finally, we investigate the alternative explanation for the pattern observed in the original study, which involves numeracy being associated with greater deference to prior factual beliefs rather than identity (4). Preregistration available at https://aspredicted.org/f7jw6.pdf.

## Results

**Fig. 1.**
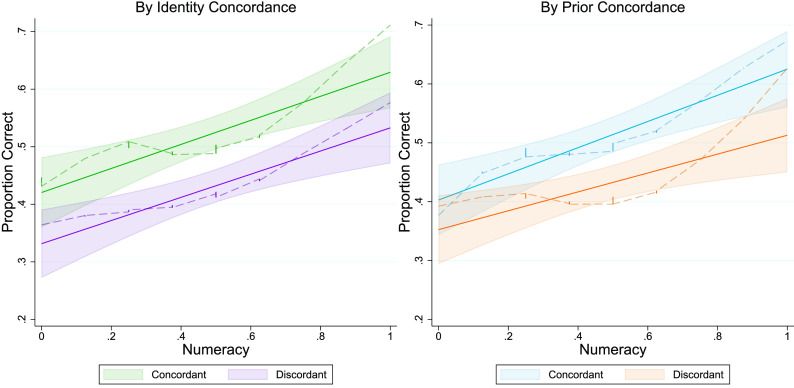
More numerate participants perform better at interpreting the contingency table, regardless of whether the correct answer aligns with their identity or prior beliefs. Shown is probability of giving the correct answer on the contingency table as a function of numeracy score, split by how the correct answer aligns with identity (*A*; green = concordant, purple = discordant) or prior beliefs (*B*; blue = concordant, orange = discordant). Solid lines indicate linear regression lines (with 95% CIs), and dotted lines indicate local polynomial regression locally estimated scatterplot smoothing line (LOESS) to show underlying data patterns.

Results are shown in Fig. 1 (see *SI Appendix* for details of our modeling approach). Examining main effects, we find that when the study’s outcome was concordant with participants’ political identity, they were 9.3 percentage points more likely to give the correct answer compared to when the outcome was discordant (*β* = 0.093, CI = [0.052, 0.135], *P* < 0.001) and that participants with higher numeracy scores were more likely to give the correct answer (*β* = 0.111, CI = [0.035, 0.077], *P* < 0.001). These results serve as manipulation checks on the identity manipulation and numeracy measure. (Note that all analyses use linear regression, and results are qualitatively equivalent when controlling for all demographics collected or when using a logistic model given the dichotomous outcome.)

Now we turn to the key test of MS2R: Does the relationship between reasoning ability and answer correctness disappear when the correct outcome is challenging to the subject’s identity? The answer here is a clear “*No*”: When the interaction between outcome concordance and numerical ability is added to the model, we find a precisely estimated null interaction (*β* = 0.002, CI = [−0.04, 0.0043], *P* = 0.94). Numeracy shows a highly significant positive relationship with answer correctness regardless of whether the outcome is concordant (*β* = 0.111, CI = [0.099, 0.317], *P* < 0.001) or discordant (*β* = 0.110, CI = [0.095, 0.307], *P* < 0.001), see Fig. 1*A*. There is also no significant three-way interaction when including the subject’s political ideology in the model (*β* = 0.025, CI = [−0.024, 0.061], *P* = 0.387), such that there is no significant interaction between outcome concordance and numerical ability for liberal Democrats (*β* = 0.006, *P* = 0.879) or conservative Republicans (*β* = −0.001, *P* = 0.999). Results are qualitatively equivalent when also including a quadratic term for numeracy.

There was little variation in the outcome-by-numeracy interaction across the five topics. Most notably, when looking specifically at gun control (as in the original study), we find a nonsignificant interaction between outcome concordance and numerical ability that is in the opposite direction from the prediction (*β* = −0.11, *P* = 0.089); among subjects who were shown the gun control scenario, those below the median on numeracy showed a significant effect of outcome concordance, *β* = 0.192, *P* = 0.001, while those above the median on numeracy did not, *β* = 0.019, *P* = 0.806. The only topic (out of five) to show an effect qualitatively similar to the original was for taxation, and even here, the interaction was not significant (*P* = 0.069). The interaction was also not significant for any of the other topics (all *ps* > 0.3). In sum, we do not find any support for the key prediction of the MS2R account.

Finally, although we did not observe the expected interaction between outcome concordance and numeracy, we nonetheless examine the consequences of controlling for prior beliefs. A model that adds the outcome’s alignment with the subject’s prior beliefs about the contingency table’s topic and does not include any interactions, shows little difference in the main effects of outcome concordance (*β* = 0.081, *P* < 0.001) and numeracy (*β* = 0.098, *P* < 0.001), while also showing that people are 6 percentage points more likely to respond correctly when the correct answer aligns with their prior beliefs (*β* = 0.061, *P* = 0.014). When adding the concordance-numeracy and priors-numeracy interactions to the model, we continue to find no significant interaction between outcome concordance and numeracy (*β* = −0.02, *P* = 0.548) and also find no significant change in the effect of numeracy based on how well the outcome aligns with the subject’s prior beliefs (*β* = 0.007, *P* = 0.801).

## Discussion

In sum, we fail to replicate the key empirical support for the influential theory of motivated system two reasoning (MS2R) in a large, representative probability sample. Instead of reasoning ability magnifying political differences, we found evidence that people who were better at reasoning were more accurate, even for highly polarized issues. Importantly, however, we also found a robust effect of identity alignment that was uneffected by either numeracy or prior beliefs. These results raise serious questions about the key empirical evidence cited as support for MS2R theory (while nonetheless supporting the existence of some form of motivated processing) and thereby call for a reconsideration of the role of analytical, deliberative reasoning in ideologically charged contexts.

## Methods

We collected *n* = 2,355 participants from the AmeriSpeak panel via an award from the Time-Sharing Experiments for the Social Sciences group (sample size was determined by the size of the award). AmeriSpeak is a nationally representative, probability-based panel based on NORC’s National Sample Frame. It is used by gold-standard survey projects in the United States and represents the level of typicality lacking in previous work. The study was conducted online in January of 2022 and took participants roughly 11 min on average to complete. The sample was probability matched and roughly reflected the overall demographics of the US population: 51.6% female, 55.7% White, non-Hispanic, median age 44 y old. Participants provided informed consent, and this study was deemed exempt by the MIT Committee on the Use of Humans as Experimental Subjects (protocol # E-3386).

Participants were randomized to one of five political topics (gun control, health care, illegal immigration, police accountability, or increased taxation). They first indicated their position on this topic, then completed a 9-item measure of numerical ability. Next, they read a description of a study that compared cities that did versus did not enact a particular policy relevant to the political issue. The results of the study were presented in a two-by-two contingency table, and participants were asked whether the results indicated that the policy increased or decreased the relevant outcome (e.g., did gun control increase or decrease gun deaths). The rows of the contingency table (and thus the correct answer) were randomized between subjects. Further, the numbers used in each cell of the contingency table were the same as those used in the original study. See *SI Appendix* for exact wording for all items.

## Supplementary Material

Appendix 01 (PDF)

## Data Availability

CSV data have been deposited in OSF (DOI: 10.17605/OSF.IO/Q2VCM) (16).
