# Supplementary material for: No association between numerical ability and politically motivated reasoning in a large US probability sample
Source: Proc Natl Acad Sci U S A. 2023 Jul 31;120(32):e2301491120. doi: 10.1073/pnas.2301491120 (PMC10410746; doi:10.1073/pnas.2301491120)
Supplement: Supplementary file 1 — Appendix 01 (PDF) [file pnas.2301491120.sapp.pdf]

# No association between numerical ability and politically motivated reasoning in a large US probability sample: SUPPLEMENTAL MATERIALS

### *Analysis Approach*

Our analyses used OLS regression predicting contingency table answer correctness as the dependent variable (0=incorrect, 1=correct). We elected to use OLS instead of logistic regression because the coefficients are more easily interpretable, particularly in the case of interactions. All models include topic (gun control, taxation, health care, police oversight, immigration) dummies.

In our first model, we included the following independent variables.

- Numeracy: Participants' fraction of correct answers on 11 items involving mathematical word problems.
- Outcome political concordance (binary): Do the results of the study support the participant's political position or the opposition's position? 0=opposition position (e.g. gun control increases crime, for liberal-Democrats), 1=own party's position (e.g. gun control decreases crime, for liberal-Democrats).

In our second model, we tested for the key MS2R prediction whereby numeracy would be positively associated with correct answers for politically aligned outcomes, but not for politically discordant outcomes. To do so, we added the 2-way interaction between numeracy and outcome political concordance.

In our third model, we added participant political identity (a composite of a 5-level partisanship item and a 7-level ideology item as per Kahan et al. 2017, each scaled to [0,1] and then averaged), and all 2-way and 3-way interactions between numeracy, outcome political concordance, and participant political identity. To examine the numeracy-concordance 2-way interactions separately by political orientation, we also created a binary political identity measure where participants below the midpoint were classified as liberal Democrats, people above the midpoint were classified as conservative Republicans, and people exactly at the midpoint were excluded.

Our final models added outcome priors concordance. Participants answered a 100 point slider scale indicating their prior belief on the relevant issue. The outcome was scored as concordant with priors if the outcome was in the same direction as the participant's prior, discordant if in the opposite direction; and excluded if the participant's prior was exactly 50.

## *Full experimental materials*

### Priors on policy

Gun Control: Which policy approach would work better to reduce crime in the United States, fewer gun control laws or more gun control laws?

[SLIDER; 0 – Increased gun control leads to more crime, 100 – Increased gun control leads to less crime]

Health care: What best represents your position on implementing universal healthcare in the US?

[SLIDER; 0 – It's negative for the health of citizens, 100 – It's positive for the health of citizens]

Immigration: What best represents your position on the effect of illegal immigration on local economy?

[SLIDER; 0 – More immigration takes jobs from US citizens, 100 – More immigration creates jobs for US citizens]

Police oversight: What best represents your position on the implementation of increased civilian oversight of police?

[SLIDER; 0 – Oversight leads to more crime, 100 – Oversight leads to less crime]

Taxation: What best represents your position on raising taxes on wealthy individuals within the US?

[SLIDER; 0 – Ultimately decreases tax revenue, 100 – Ultimately increases tax revenue]

### NUMERACY ITEMS AND INSTRUCTIONS

In the following section you will be asked several questions. Please do your best to answer them as accurately as possible on your own and without looking up the answers. Please just do the best you can.

Q1. Imagine that we roll a fair, six-sided die 1000 times. Out of 1000 rolls, how many times do you think the die would come up as an even number?

[NUMBOX; 0-1000] times

Q2. In the BIG BUCKS LOTTERY, the chances of winning a $10.00 prize are 1%. What is your best guess about how many people would win a $10.00 prize if 1000 people each buy a single ticket from BIG BUCKS?

[NUMBOX; 0-1000] people

Q3. In the ACME PUBLISHING SWEEPSTAKES, the chance of winning a car is 1 in 1000. What percent of tickets of ACME PUBLISHING SWEEPSTAKES win a car?

[NUMBOX; 0-100] %

Q4. If the chance of getting a disease is 20 out of 100, this would be the same as having a ___% chance of getting the disease.

[NUMBOX; 0-100] %

Q5. If the chance of getting a disease is 10%, how many people would be expected to get the disease out of 1000?

[NUMBOX; 0-1000] people

Q6. Suppose your friend just had a mammogram. The doctor knows from previous studies that, of 100 women like her, 10 have tumors and 90 do not. Of the 10 who do have tumors, the mammogram correctly finds 9 with tumors and incorrectly says that 1 does not have a tumor. Of the 90 women without tumors, the mammogram correctly finds 80 without tumors and incorrectly says that 10 have tumors. The table below summarizes this information. Imagine that your friend tests positive (as if she had a tumor), what is the likelihood that she actually has a tumor?

[SPACE]

| | **Tested Positive** | **Tested Negative** | **Totals** |
|---|---|---|---|
| **Actually has a tumor** | 9 | 1 | 10 |
| **Does not have a tumor** | 10 | 80 | 90 |
| **Totals** | 19 | 81 | 100 |

[NUMBOX; 0-100] out of [NUMBOX; 0-100]

Q7. If John can drink one jug of water in 6 days, and Mary can drink one jug of water in 12 days, how long would it take them to drink one jug of water together?

[NUMBOX; 0-10] days

Q8. A sandwich and a cookie cost $5.50. The sandwich costs $5 more than the cookie. How much does the cookie cost?

[NUMBOX; 0-550] ₵

Q9. In a field, there is a colony of mushrooms. Every day, the colony doubles in size. If it takes 28 days for the colony to cover the entire field, how long would it take for the colony to cover half of the field?

[NUMBOX; 0-28] days

**CONDITION CONTENT AND OUTCOME FRAME**

[Condition 1: Gun Control = decreases crime]

Please read the following description of an experiment that looked at gun bans and crime and do your best to answer the question below.

A city government is trying to decide whether to pass a law banning private citizens from carrying concealed handguns in public. Government officials are unsure whether the law will be more likely to decrease crime by reducing the number of people carrying weapons or increase crime by making it harder for law-abiding citizens to defend themselves from violent criminals.

To address this question, researchers divided cities into two groups: one consisting of cities that had recently enacted bans on concealed weapons, and another that had no such bans. They then observed the number of cities that experienced decreases in crime and the number of cities that experienced increases in crime in the next year.

In each group, the number of cities that experienced a decrease in crime and the number of cities that experienced an increase in crime is recorded below. The total number of cities in each of the two groups is not exactly the same, but this does not prevent assessment of the results.

Please indicate whether the research shows that cities that enacted a ban on carrying concealed handguns were more likely to have a decrease or an increase in crime than cities without bans.

| Results | Increase in crime | Decrease in crime |
|---|---|---|
| Cities that did ban carrying concealed handguns in public | 223 | 75 |
| Cities that did not ban carrying concealed handguns in public | 107 | 21 |

What result does the study support?

[RANDOMIZE RESPONSE OPTIONS]

1. Cities that enacted a ban on carrying concealed handguns were more likely to have a decrease in crime than cities without bans.

2. Cities that enacted a ban on carrying concealed handguns were more likely to have an increase in crime than cities without bans.

[Condition 2: Gun Control = increases crime]

Please read the following description of an experiment that looked at gun bans and crime and do your best to answer the question below.

A city government is trying to decide whether to pass a law banning private citizens from carrying concealed handguns in public. Government officials are unsure whether the law will be more likely to decrease crime by reducing the number of people carrying weapons or increase crime by making it harder for law-abiding citizens to defend themselves from violent criminals.

To address this question, researchers divided cities into two groups: one consisting of cities that had recently enacted bans on concealed weapons, and another that had no such bans. They then observed the number of cities that experienced decreases in crime and the number of cities that experienced increases in crime in the next year.

In each group, the number of cities that experienced a decrease in crime and the number of cities that experienced an increase in crime is recorded below. The total number of cities in each of the two groups is not exactly the same, but this does not prevent assessment of the results.

Please indicate whether the research shows that cities that enacted a ban on carrying concealed handguns were more likely to have a decrease or an increase in crime than cities without bans.

| Results | Decrease in crime | Increase in crime |
|---|---|---|
| Cities that did ban carrying concealed handguns in public | 223 | 75 |
| Cities that did not ban carrying concealed handguns in public | 107 | 21 |

What result does the study support?

[RANDOMIZE RESPONSE OPTIONS]

1. Cities that enacted a ban on carrying concealed handguns were more likely to have a decrease in crime than cities without bans.

2. Cities that enacted a ban on carrying concealed handguns were more likely to have an increase in crime than cities without bans.

[Condition 3: Taxation = decreases funds]

Please read the following depiction of an experiment that looks at taxation laws and city funds and do your best to answer the question below.

A city government is trying to decide whether to pass a law increasing taxes on residents earning over $250k a year. Government officials are unsure whether the law will be more likely to decrease city funds by driving high earning residents out of the city or increase city funds by securing more taxes from those high earners who stay.

To address this question, researchers divided cities into two groups: one consisting of cities that had recently enacted a tax increase on residents earning over $250k, and another that had no such tax increase. They then observed the number of cities that experienced decreases in city funds and the number of cities that experienced increases in city funds in the next year.

In each group, the number of cities that experienced a decrease in funds and the number of cities that experienced an increase in funds is recorded below. The total number of cities in each of the two groups is not exactly the same, but this does not prevent assessment of the results.

Please indicate whether the research shows that cities that enacted a tax increase on high earning residents were more likely to have a decrease or an increase in funds than cities without a tax increase.

| <u>Results</u> | Increase in funds | Decrease in funds |
|---|---|---|
| Cities that <u>did</u> have a tax increase on high earning residents | 223 | 75 |
| Cities that <u>did not</u> have a tax increase on high earning residents | 107 | 21 |

What result does the study support?

[RANDOMIZE RESPONSE OPTIONS]

1. Cities that enacted a tax increase on high earning residents were more likely to have a decrease in funds than cities without a tax increase.

2. Cities that enacted a tax increase on high earning residents were more likely to have an increase in funds than cities without a tax increase.

[Condition 4: Taxation = increases funds]

Please read the following depiction of an experiment that looks at taxation laws and city funds and do your best to answer the question below.

A city government is trying to decide whether to pass a law increasing taxes on residents earning over $250k a year. Government officials are unsure whether the law will be more likely to decrease city funds by driving high earning residents out of the city or increase city funds by securing more taxes from those high earners who stay.

To address this question, researchers divided cities into two groups: one consisting of cities that had recently enacted a tax increase on residents earning over $250k, and another that had no such tax increase. They then observed the number of cities that experienced decreases in city funds and the number of cities that experienced increases in city funds in the next year.

In each group, the number of cities that experienced a decrease in funds and the number of cities that experienced an increase in funds is recorded below. The total number of cities in each of the two groups is not exactly the same, but this does not prevent assessment of the results.

Please indicate whether the research shows that cities that enacted a tax increase on high earning residents were more likely to have a decrease or an increase in funds than cities without a tax increase.

| Results | Decrease in funds | Increase in funds |
|---|---|---|
| Cities that did have a tax increase on high earning residents | 223 | 75 |
| Cities that did not have a tax increase on high earning residents | 107 | 21 |

What result does the study support?

[RANDOMIZE RESPONSE OPTIONS]

1. Cities that enacted a tax increase on high earning residents were more likely to have a decrease in funds than cities without a tax increase.

2. Cities that enacted a tax increase on high earning residents were more likely to have an increase in funds than cities without a tax increase.

[Condition 5: Health care = decreases public health]

Please read the following depiction of an experiment that looks at government run healthcare and overall public health and do your best to answer the question below.

A city government is trying to decide whether to pass a law centralizing all healthcare, making it tax-funded and city-run. Government officials are unsure whether the law will be more likely to decrease overall public health by removing choices and competition in the healthcare market or increase overall public health by decreasing costs while increasing access to covered services.

To address this question, researchers divided cities into two groups: one consisting of cities that had recently enacted tax-funded centralized healthcare laws, and another that had no such laws. They then observed the number of cities that experienced decreases in overall public health and the number of cities that experienced increases in overall public health in the next year.

In each group, the number of cities that experienced a decrease in overall public health and the number of cities that experienced an increase in overall public health is recorded

below. The total number of cities in each of the two groups is not exactly the same, but this does not prevent assessment of the results.

Please indicate whether the research shows that cities that enacted a government run healthcare law were more likely to have a decrease or an increase in overall public health than cities without such a law.

| Results | Increase in overall public health | Decrease in overall public health |
|---|---|---|
| Cities that did enact a government run healthcare law | 223 | 75 |
| Cities that did not enact a government run healthcare law | 107 | 21 |

What result does the study support?

[RANDOMIZE RESPONSE OPTIONS]

1. Cities that enact a government run healthcare law were more likely to have a decrease in overall public health than cities without such a law.

2. Cities that enact a government run healthcare law were more likely to have an increase in overall public health than cities without such a law.

[Condition 6: Health care = increases public health]

Please read the following depiction of an experiment that looks at government run healthcare and overall public health and do your best to answer the question below.

A city government is trying to decide whether to pass a law centralizing all healthcare, making it tax-funded and city-run. Government officials are unsure whether the law will be more likely to decrease overall public health by removing choices and competition in the healthcare market or increase overall public health by decreasing costs while increasing access to covered services.

To address this question, researchers divided cities into two groups: one consisting of cities that had recently enacted tax-funded centralized healthcare laws, and another that had no such laws. They then observed the number of cities that experienced decreases in overall public health and the number of cities that experienced increases in overall public health in the next year.

In each group, the number of cities that experienced a decrease in overall public health and the number of cities that experienced an increase in overall public health is recorded below. The total number of cities in each of the two groups is not exactly the same, but this does not prevent assessment of the results.

Please indicate whether the research shows that cities that enacted a government run healthcare law were more likely to have a decrease or an increase in overall public health than cities without such a law.

| <u>Results</u> | Decrease in overall public health | Increase in overall public health |
|---|---|---|
| Cities that <u>did</u> enact a government run healthcare law | 223 | 75 |
| Cities that <u>did not</u> enact a government run healthcare law | 107 | 21 |

What result does the study support?

[RANDOMIZE RESPONSE OPTIONS]

1. Cities that enact a government run healthcare law were more likely to have a decrease in overall public health than cities without such a law.

2. Cities that enact a government run healthcare law were more likely to have an increase in overall public health than cities without such a law.

[Condition 7: Police oversight = decrease in crime]

Please read the following depiction of an experiment that looks at policing regulation and crime and do your best to answer the question below.

A city government is trying to decide whether to pass a law allowing more civilian oversight and regulation of the police. Government officials are unsure whether the law will be more likely to decrease crime by holding officers accountable and signaling more support for underserved communities or increase crime by making it harder for police to carry out regular duties and to hire more officers overall.

To address this question, researchers divided cities into two groups: one consisting of cities that had recently enacted civilian oversight laws, and another that had no such laws. They then observed the number of cities that experienced decreases in crime and the number of cities that experienced increases in crime in the next year.

In each group, the number of cities that experienced a decrease in crime and the number of cities that experienced an increase in crime is recorded below. The total number of cities in each of the two groups is not exactly the same, but this does not prevent assessment of the results.

Please indicate whether the research shows that cities that enacted a civilian oversight law for police were more likely to have a decrease or an increase in crime than cities without such a law.

| <u>Results</u> | Increase in crime | Decrease in crime |
|---|---|---|
| Cities that <u>did</u> enact a civilian oversight law for police | 223 | 75 |
| Cities that <u>did not</u> enact a civilian oversight law for police | 107 | 21 |

What result does the study support?

[RANDOMIZE RESPONSE OPTIONS]

1. Cities that enacted a civilian oversight law for police were more likely to have a decrease in crime than cities without such a law.

2. Cities that enacted a civilian oversight law for police were more likely to have an increase in crime than cities without such a law.

[Condition 8: Police oversight = increase in crime]

Please read the following depiction of an experiment that looks at policing regulation and crime and do your best to answer the question below.

A city government is trying to decide whether to pass a law allowing more civilian oversight and regulation of the police. Government officials are unsure whether the law will be more likely to decrease crime by holding officers accountable and signaling more support for underserved communities or increase crime by making it harder for police to carry out regular duties and to hire more officers overall.

To address this question, researchers divided cities into two groups: one consisting of cities that had recently enacted civilian oversight laws, and another that had no such laws. They then observed the number of cities that experienced decreases in crime and the number of cities that experienced increases in crime in the next year.

In each group, the number of cities that experienced a decrease in crime and the number of cities that experienced an increase in crime is recorded below. The total number of cities in each of the two groups is not exactly the same, but this does not prevent assessment of the results.

Please indicate whether the research shows that cities that enacted a civilian oversight law for police were more likely to have a decrease or an increase in crime than cities without such a law.

| Results | Decrease in crime | Increase in crime |
|---|---|---|
| Cities that did enact a civilian oversight law for police | 223 | 75 |
| Cities that did not enact a civilian oversight law for police | 107 | 21 |

What result does the study support?

[RANDOMIZE RESPONSE OPTIONS]

1. Cities that enacted a civilian oversight law for police were more likely to have a decrease in crime than cities without such a law.

2. Cities that enacted a civilian oversight law for police were more likely to have an increase in crime than cities without such a law.

[Condition 9: Immigration = decrease in local employment]

Please read the following depiction of an experiment that looks at immigrant sanctuary cities and local employment and do your best to answer the question below.

A city government is trying to decide whether to pass a law that would designate the city a “sanctuary city” and resist the deportation of non-violent illegal immigrants. Government officials are unsure whether the law will be more likely to decrease employment rates of local residents by creating more competition for jobs with a cheaper labor supply or increase employment rates of local residents by growing the local economy and creating new jobs.

To address this question, researchers divided cities into two groups: one consisting of cities that had recently enacted sanctuary city laws, and another that had no such laws. They then observed the number of cities that experienced decreases in local resident

employment and the number of cities that experienced increases in local resident employment in the next year.

In each group, the number of cities that experienced a decrease in local resident employment and the number of cities that experienced an increase in local resident employment is recorded below. The total number of cities in each of the two groups is not exactly the same, but this does not prevent assessment of the results.

Please indicate whether the research shows that cities that enacted sanctuary city laws were more likely to have a decrease or an increase in local resident employment than cities without such laws enacted.

| Results | Increase in local resident employment | Decrease in local resident employment |
|---|---|---|
| Cities that did enact sanctuary city laws | 223 | 75 |
| Cities that did not enact sanctuary city laws | 107 | 21 |

What result does the study support?

[RANDOMIZE RESPONSE OPTIONS]

1. Cities that enacted sanctuary city laws were more likely to have a decrease in local resident employment than cities without such laws.

2. Cities that enacted sanctuary city laws were more likely to have an increase in local resident employment than cities without such laws.

[Condition 10: Immigration = increase in local employment]

Please read the following depiction of an experiment that looks at immigrant sanctuary cities and local employment and do your best to answer the question below.

A city government is trying to decide whether to pass a law that would designate the city a “sanctuary city” and resist the deportation of non-violent illegal immigrants. Government officials are unsure whether the law will be more likely to decrease employment rates of local residents by creating more competition for jobs with a cheaper labor supply or

increase employment rates of local residents by growing the local economy and creating new jobs.

To address this question, researchers divided cities into two groups: one consisting of cities that had recently enacted sanctuary city laws, and another that had no such laws. They then observed the number of cities that experienced decreases in local resident employment and the number of cities that experienced increases in local resident employment in the next year.

In each group, the number of cities that experienced a decrease in local resident employment and the number of cities that experienced an increase in local resident employment is recorded below. The total number of cities in each of the two groups is not exactly the same, but this does not prevent assessment of the results.

Please indicate whether the research shows that cities that enacted sanctuary city laws were more likely to have a decrease or an increase in local resident employment than cities without such laws enacted.

| Results | Decrease in local resident employment | Increase in local resident employment |
|---|---|---|
| Cities that did enact sanctuary city laws | 223 | 75 |
| Cities that did not enact sanctuary city laws | 107 | 21 |

What result does the study support?

[RANDOMIZE RESPONSE OPTIONS]

1. Cities that enacted sanctuary city laws were more likely to have a decrease in local resident employment than cities without such laws.

2. Cities that enacted sanctuary city laws were more likely to have an increase in local resident employment than cities without such laws.
